# Clinical significance of the uPA system in gastric cancer with peritoneal metastasis

**DOI:** 10.1186/2047-783X-18-28

**Published:** 2013-08-28

**Authors:** Youcheng Ding, Hui Zhang, Mingan Zhong, Zhuqing Zhou, Zhixiang Zhuang, Hua Yin, Xujing Wang, Zhenggang Zhu

**Affiliations:** 1 Department of General Surgery, East Hospital, TongJi University School of Medicine, 150 Jimo Road, Shanghai 200120, China; 2 Department of Gastroenterology, Ruijin Hospital, Shanghai Jiao Tong University School of Medicine, 197 Ruijin Road II, Shanghai 200025, China

**Keywords:** Gastric cancer, ELISA, Peritoneal metastasis, RT-PCR, UPA system

## Abstract

**Background:**

It has been demonstrated that urokinase-type plasminogen activator (uPA) is involved in tumor cell metastasis by degrading the extracellular matrix. However, there is little direct evidence of clinical uPA system expression in peritoneal metastatic tissues of gastric cancer. The objective of this study was to investigate uPA system expression in peritoneal tissues of peritoneal and nonperitoneal metastasis patients, and to explore the diagnostic value of the uPA system.

**Methods:**

Expressions of uPA, uPAR, and PAI-1 were measured by semi-quantitative RT-PCR and ELISA. uPA activity was detected using a uPA activity kit.

**Results:**

There was no significant difference in uPA, uPAR, and PAI-1 expression in two types of peritoneal tissue in seven patients with peritoneal metastasis. However, uPA, uPAR, and PAI-1 expressions in peritoneal metastatic lesions were significantly higher than those in normal peritoneal tissues of 24 nonperitoneal metastasis patients (*P* <0.05). Moreover, no statistical discrepancy of uPA activity was observed in various different tissues.

**Conclusions:**

The expression of the uPA system positively correlates with peritoneal metastasis of gastric cancer. This expression difference in peritoneal or nonperitoneal metastasis patients may provide a reference for diagnosis of peritoneal metastasis.

## Background

Although the incidence and mortality of gastric cancer have decreased in China over the last few decades, gastric cancer is still a big burden of the local health program [1]. Importantly, the recurrence of gastric cancer occurs even after potentially curative resection, most frequently in the form of peritoneal metastasis [2]. Thus, there is an urgent need to develop effective early diagnosis strategies for peritoneal metastasis of gastric cancer.

Cancer invasion and metastasis are multifactorial processes [3] and an essential step involves consecutive destruction and reconstitution of the extracellular matrix and basement membranes, which in turn requires the participation of several proteolytic enzyme systems, such as serine proteases and metalloproteases [4]. Urokinase-type plasminogen activator (uPA) is one of the serine proteinases and it binds to its receptor, the uPA receptor (uPAR) on the surface of the tumor cell. After activation, cell-bound uPA is capable of converting plasminogen into plasmin, which is then able to degrade several components of the extracellular matrix [5]. The action of the uPA-uPAR complex on plasminogen can be controlled by the protease inhibitors PAI [6,7]. Thus, a balanced production of cellular and pericellular uPA, uPAR, and PAI-1 is the prerequisite for efficient focal proteolysis, cell adhesion, and migration, and hence, tumor cell invasion and metastasis [8,9].

It has been observed that the uPA system is well correlated with gastric cancers, by measuring the expression level of uPA, uPAR, and PAI-1 in gastric cancer and normal mucosal tissue and analyzing their correlation with various clinical pathological characteristics. For example, Plebani *et al*. [10] determined uPAR, uPA, and PAI-1 levels using ELISA in gastric cancer and normal samples from 20 patients with gastric cancer undergoing surgery. uPAR expression level is significantly higher in gastric cancer, and low levels of uPAR are associated with a better survival. Taniguchi *et al*. [11] studied the relationship between the expression of uPAR and clinicopathologic parameters using immunohistochemical analysis from 102 primary gastric carcinomas. uPAR immunoreactivity was observed in 38 cases out of 102 (37%). In addition, the expressions of uPA, uPAR, and PAI-1 are significantly correlated with various clinicopathological factors: tumor size, depth of tumor invasion, differentiation, lymph node metastasis [12-15], and peritoneum metastasis [16,17].

However, there has been very little direct evidence to demonstrate the clinical significance of the uPA system in distinguishing peritoneal metastatic from normal peritoneal tissues in gastric cancer. Compared with the regular distribution and arrangement of lymph nodes, peritoneal tissue has a larger area, occupying the whole peritoneal cavity, thus entailing more randomness and unpredictability in cell peritoneal metastasis in gastric cancer. Thus, the objective of this study was to investigate further the expression difference of the uPA system between peritoneal metastatic tissues and normal peritoneal tissues of gastric cancer and to confirm the diagnosis significance of the uPA system by combining these results with clinical data.

## Methods

### Clinical sample

We assessed 31 patients (21 men, 10 women) with a diagnosis of gastric cancer, who had been admitted to our department between July 2010 and December 2010. Their average age was 62.58 years (range, 23 to 85). The patients were diagnosed with gastric cancer based on the preoperative or postoperative pathological analysis. Among them, 7 patients had peritoneal metastasis (pathological type: 1 case of moderately differentiated tubular adenocarcinoma, 1 case of grade II to III adenocarcinoma, 3 cases of poorly differentiated adenocarcinoma, and 3 cases of poorly differentiated adenocarcinoma combined with signet ring cell carcinoma), while 24 patients had nonperitoneal metastasis (pathological type: 3 cases of moderately differentiated tubular adenocarcinoma, 3 cases of grade II adenocarcinoma, 2 cases of grade II to III adenocarcinoma, 9 cases of poorly differentiated adenocarcinoma, 3 cases of signet ring cell carcinoma, 1 case of mucinous carcinoma, 1 case of grade II adenocarcinoma combined with signet ring cell carcinoma, 1 case of grade III adenocarcinoma combined with signet ring cell carcinoma, and 1 case of grade II adenocarcinoma combined with mucinous carcinoma).

In the patients with peritoneal metastasis, we excised the peritoneal metastasis lesions, as well as a small amount of omentum majus, pelveoperitoneum, and diaphragmatic peritoneum without peritoneal metastasis. In the patients with nonperitoneal metastasis, a few resections of the omentum majus, pelveoperitoneum, and diaphragmatic peritoneum were also performed. The collected samples were stored at −80°C for further analysis.

All patients were alive at the end of the research, and our study was approved by the ethics committee of Shanghai East Hospital, affiliated to Tong Ji University.

### Cell culture

Cells from a peritoneal mesothelial cell line (HMrSV5) were maintained in DMEM (Sigma, St. Louis, USA) supplemented with 10% fetal bovine serum. The cells were subcultured almost every day by 1:4 or 1:5 dilution in culture flasks at 37°C under an atmosphere of 5% CO_2_.

### Total RNA extraction and cDNA synthesis

Total RNA was isolated by cell precipitation using an RNeasy™ RNA extraction kit following the manufacturer’s instructions (Qiagen, Valencia, USA). The RNA purity and concentration were determined by spectrophotometric absorbance at 260 and 280 nm, respectively. Reverse transcription was performed on 1 μg of total RNA using oligo(dT) primers and AMV reverse transcriptase (Promega, Madison, USA). The 20 μl PCR reaction mixture contained 2 μl 10× RT buffer, 2 μl dNTPs, 4 μl MgCl_2_, 0.5 μl RNasin, 1 μl oligo(dT)_18_, 0.75 μl reverse transcriptase, and 1 μg RNA. The PCR condition was 70°C for 10 min, 42°C for 15 min, and 99°C for 5 min, after which the cDNA was stored at 4°C (within 3 months).

### Semi-quantitative RT-PCR

The gene expression levels of uPA, uPAR, and PAI-1 were determined by comparison with β-actin gene or glyceraldehyde-3-phosphate dehydrogenase (GAPDH). The 25 μl RT-PCR reaction mixture contained 1 μl cDNA, sense and anti-sense primers (each 0.25 μl), 2.5 μl 10× PCR buffer, 2 μl 25% MgCl_2_, 0.5 μl Taq polymerase, 1 μl dNTP, and 17.5 μl Rnase-free H_2_O. Nested PCR was used for the amplification of carcinoembryonic antigen (CEA) with 1 μl of first PCR product used for the second PCR template. The amplification conditions were: (i) initial denaturation at 95°C for 5 min; (ii) 30 cycles of denaturation at 94°C for 1 min; (iii) annealing at 56°C for 30 s for uPA, 60°C for 1 min for uPAR, 55°C for 1 min for PAI-1, or 72°C for 2.5 min for CEA; and (iv) extension at 72°C for 1 min or 2.5 min. The PCR primers used were as follows: for uPA, A, 5′-AGAATTCACCACCATCGA GA-3′ and B, 5′-ATCAGCTTCACAACAGTCA T-3′; for uPAR, A, 5′-ACA GGAGCTGCCCTCGCGAC-3′ and B, 5′-GAGGGGGATTT CAGGTTT AGG-3′; for PAI-1, A, 5′-CTTTGGTGAAGGGTCTGC-3′ and B, 5′-CTC CACCTCTGAAAAGTCC-3′; for CEA, A, 5′-TCTGGAACTTCTCCTGGTCT CAGCTGG-3′, B, 5′-TGTAGCTGTTGCAAATGCTTTAAGGAAGAAGC-3′, and C, 5′-GGGCCACTGTCGGCATCATG ATTGG-3′; for β-actin, A, 5′-TTGAAGGTAGTTTC GGGAAT-3′ and B, 5′-GAA AATCTG GCACCACAC CTT-3′; for GAPDH, A, 5′-GAAGGTGAAGGCGGAGT C-3′ and B, 5′-GAAGATGGTGATGGGATTTC-3′. The A and B primers of CEA were used for the first PCR, while the B and C primers were used for the second amplification. The amplification product was 474 bp, 1046 bp, 409 bp, 131 bp, 591 bp, and 230 bp, respectively.

### Quantitative ELISA analysis

Tris-buffered saline (pH 8.5, 1.8 ml) was added to frozen tissues (100 to 300 mg) and homogenization was performed in an ice-bath. Subsequently, 0.2 ml 10% Trixon X-100 was added to ensure a final concentration of 1% Trixon X-100 in the homogenate. The homogenate was shaken for 16 h and then centrifuged in a refrigerated centrifuge at 4°C for 1 h at 100,000 *g*. The supernatant was transferred to a new tube and the protein concentration was determined by a bicinchoninic acid assay. Concentrations of uPA, uPAR, and PAI-1 antigen were determined using ELISA kits according to the manufacturers’ instructions (American Diagnostica, Greenwich, USA). The reaction was stopped by the addition of 50 μl H_2_SO_4_, and the absorption was measured at 450 nm on an ELISA plate reader (EL312e microplate reader, Bio-Tek Instruments, Winooski, USA). Values of uPA, uPAR, and PAI-1 antigen were expressed as ng/mg protein.

### uPA activity assay

uPA activity was measured using an uPA activity assay kit (Chemicon). Briefly, tissue protein was mixed with assay buffer and incubated with a chromogenic substrate in 96-well plates at 37°C for 2 to 24 h. The absorbance was read at OD_405_, and the activity (units) was extrapolated from a standard curve.

### Statistical analysis

All data were analyzed using SAS version 6.12 software and the results were recorded as average ± standard deviation. The significance of the differences between groups was evaluated by analysis of variance, followed by a paired *t* test. *P* < 0.05 was considered statistically significant.

## Results

### Semi-quantitative RT-PCR analysis of CEA, uPA, uPAR, and PAI-1 mRNA expression

**Figure 1:**
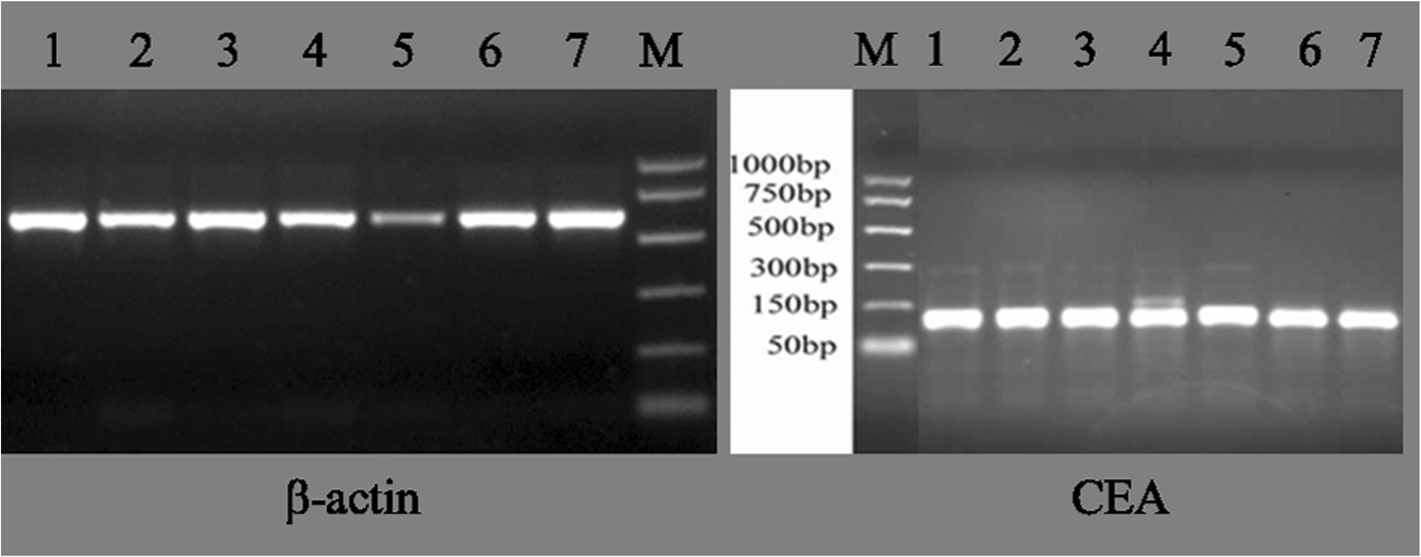
**CEA mRNA expression in seven cases of gastric cancer with peritoneal metastasis.** M: 1000 bp marker; Lanes 1, 2, 3, 4, 5, 6, and 7 indicated the seven peritoneal metastatic cases. β-actin was used for internal reference to normalize the expression of CEA. The amplification products were 131 bp of CEA and 591 bp of β-actin, respectively. CEA, carcinoembryonic antigen.

**Figure 2:**
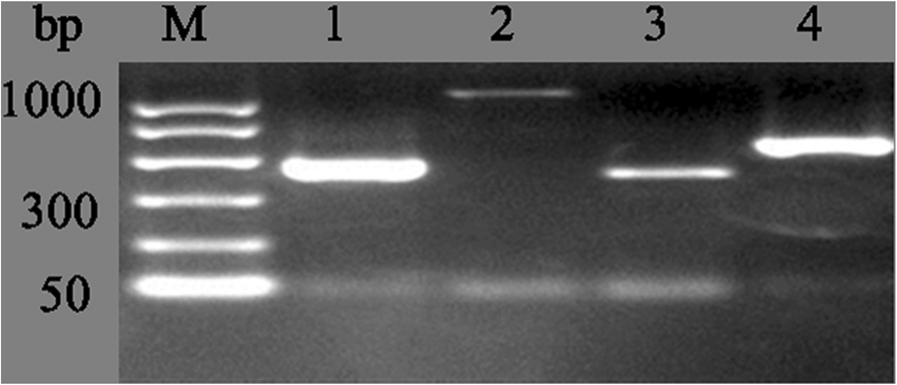
**uPA system mRNA expression in HMrSV5 cell.** M, 1000 bp marker; lane 1, uPA; lane 2, uPAR; lane 3, PAI-1; lane 4, β-actin. β-actin was used for internal reference. The amplification products were 474 bp of uPA, 1046 bp of uPAR, 409 bp of PAI-1, and 591 bp of β-actin, respectively.

CEA is an important marker for gastroenteric tumors. Thereby, we used a high sensibility nested RT-PCR method to detect the expression of CEA in gastric cancer tissues with or without peritoneal metastasis. As expected, the results were all positive for CEA in the peritoneal metastatic lesions of seven peritoneal metastasis patients (Figure 1). Also, by naked eye observation of peritoneal metastasis patients, three cases showed CEA-positive expression in the nonperitoneal metastatic tissues. Among the 24 patients with nonperitoneal metastasis, 2 had CEA-positive expression in normal peritoneal tissues. No CEA expression was detected in the peritoneal mesothelial cell line HMrSV5. However, uPA, uPAR, and PAI-1could be expressed in HMrSV5 cells (Figure 2).

### uPA system protein expression in gastric cancer tissues with or without peritoneal metastasis

A quantitative ELISA method was used to determinate the uPA, uPAR, and PAI-1 protein contents in peritoneal metastatic lesions and CEA-negative nonperitoneal metastatic tissues of 7 peritoneal metastasis patients, and CEA-negative normal peritoneal tissues of 24 nonperitoneal metastasis patients. The results (Table 1) indicated that there were no significant differences in uPA, uPAR, and PAI-1 expression in two types of peritoneal tissue of seven peritoneal metastasis patients. However, uPA, uPAR, and PAI-1 expression were significantly higher in peritoneal metastatic lesions than those normal peritoneal tissues of 24 nonperitoneal metastasis patients (*P* < 0.05).

**Table 1:** uPA system protein expression in gastric cancer tissues with or without peritoneal metastasis

**Protein**	**Peritoneal metastasis (7 cases)**		**Nonperitoneal metastasis (24 cases)**		
	**CEA(+)**	**CEA(−)**	**CEA(−)**		
uPA	3.312	2.488	0.408	0.784	0.640
	3.088	1.664	0.280	0.632	0.488
	2.952	0.648	0.672	0.664	0.224
	0.712	0.504	0.280	0.800	0.424
	0.520	0.488	0.440	0.656	0.328
	0.480	0.128	0.440	0.256	0.384
	0.376	1.440	0.512	0.440	0.848
			0.312	0.464	0.168
uPAR	3.664	4.936	1.432	0.345	1.034
	2.720	2.304	0.640	1.256	0.532
	2.744	2.192	1.536	0.239	0.479
	1.432	1.808	0.704	0.671	0.561
	0.912	1.280	0.792	0.845	0.390
	1.184	0.480	0.272	0.432	0.633
	1.064	2.728	1.280	0.467	0.291
			1.120	0.968	0.776
PAI-1	1.511	4.204	0.568	3.665	No detection
	3.872	1.725	0.262	1.426	0.071
	2.782	0.876	0.488	3.598	No detection
	0.369	0.745	0.662	0.488	No detection
	1.315	3.023	0.894	0.127	0.289
	1.041	0.262	0.963	No detection	0.195
	1.181	0.977	0.041	0.329	0.395
			0.085	0.316	0.382

### uPA activity detection

We also detected uPA activity in peritoneal metastatic lesions and CEA-negative nonperitoneal metastatic tissues of 7 peritoneal metastasis patients, and CEA-negative normal peritoneal tissues of 24 nonperitoneal metastasis patients. The results indicated that no statistical discrepancy was observed in various different tissues (Table 2).

**Table 2:** uPA activity in gastric cancer tissues with or without peritoneal metastasis

**Peritoneal metastasis (7 cases)**		**Nonperitoneal metastasis (24 cases)**		
**CEA(+)**	**CEA(−)**	**CEA(−)**		
19.936	16.536	3.914	4.510	5.635
0.846	3.091	1.942	5.407	4.151
1.417	8.725	3.117	4.702	0.899
2.352	16.170	2.142	10.046	2.419
2.820	4.799	1.302	3.730	2.828
2.123	3.310	5.299	7.287	1.233
6.066	6.533	7.922	6.485	8.150
		7.802	4.466	3.468

## Discussion

Owing to its lack of specific clinical manifestations in the early stage of peritoneal metastasis, the optimal treatment opportunity has always been missed in gastric cancer patients. The occurrence of ascites, abdominal tumors, and intestinal obstructions indicates an advanced stage of gastric cancer. Therefore, the effective diagnosis of gastric cancer with peritoneal metastasis is still a challenge in clinics. Currently, the main diagnostic method for gastric cancer with peritoneal metastasis includes peritoneal lavage and cytological investigation [18,19]. However, positive results only indicate subclinical peritoneal metastasis because the peritoneal metastasis is also not emergent even if tumor cells are present in the peritoneal fluid. Thus, detection in peritoneal tissues seems to be more direct and accurate. Remarkably, peritoneal tissues cover the whole peritoneal cavity, while tumor cell implantation is random, so direct detection of tumor cells in peritoneal tissues is very difficult. Molecular biologists believe that there are some molecular changes in self-tumor cells and their affected tissues, and these changes in tissues may be initiated before the tumor cells contact them [9]. Based on this concept, we investigated molecular changes in peritoneal tissues to explore the potential diagnosis of peritoneal metastasis.

CEA is a common tumor-associated antigen, and is accepted internationally as the gastroenteric tumor marker [20,21]. Therefore, we detected the expression of CEA in peritoneal tissues. Positive CEA-expression results indicate that tumor cells are present in peritoneal tissues. As expected, our results showed that CEA was expressed in all the peritoneal metastatic lesions of seven peritoneal metastasis patients. Interestingly, there were three patients with peritoneal metastasis in whom CEA was positively expressed in the nonperitoneal metastatic tissues and two patients with nonperitoneal metastasis who had positive CEA expression in normal peritoneal tissues. This suggests that these patients had a potential for developing peritoneal metastasis.

As it includes important proteolytic enzymes, the uPA system in tumor cells has been demonstrated to interact with the extracellular matrix to facilitate the microenvironment formation of metastases; thus, the uPA system may be involved in the process of peritoneal metastasis [22]. Expression of the uPA system is found to be increased in implanted tumor and host tissues [23,24]. The uPA system consists of the serine protease uPA, the glycolipid-anchored receptor, uPAR, and the serpin inhibitor, PAI-1, [25]. Therefore, we aimed to investigate comprehensively changes in these three factors in peritoneal tissues with or without metastasis.

The uPA system is widely distributed in various cells; our RT-PCR showed that uPA, uPAR, and PAI-1 could even be expressed in mesothelial cells. To avoid unspecific results, further quantitative ELISA was used to detect expression of the uPA system proteins. We found that uPA, uPAR, and PAI-1 protein contents were significantly higher in CEA-positive lesions or CEA-negative peritoneal tissues of peritoneal metastasis patients when compared with normal peritoneal tissues of nonperitoneal metastasis patients (*P* < 0.05). Notably, there may be greater clinical significance in the increased uPA system protein content in CEA-negative peritoneal tissues of peritoneal metastasis patients. This finding indicated that these CEA-negative peritoneal tissues might be in a state of subclinical peritoneal metastasis and that progression of this disease might lead to peritoneal metastasis. Although uPA system changes in peritoneal tissues have not yet definitely been demonstrated in tumor cells, the changes in peritoneal tissues at least provides a reference value for diagnosis of peritoneal metastasis [26]. Heiss *et al*. [27] find that uPAR could be considered as a dependent index for prediction of stomach cancer bone marrow micrometastasis compared with cytokeratin (CK18). In this study, uPAR protein content was also significantly higher in CEA-negative peritoneal tissues of peritoneal metastasis patients than in CEA-negative peritoneal tissues of nonperitoneal metastasis patients (*P* < 0.05). In brief, we suggest that increased uPAR and PAI-1 expressions in peritoneal tissues of nonperitoneal metastasis patients may be regarded as a reference indicator for peritoneal metastasis.

By assay of the uPA activity in gastric cancer tissues and their extracellular matrix, Okusa *et al*. [28] report that the higher uPA activity is significantly associated with tumors with peritoneal metastases and tumors with deeper invasion into the gastric wall. uPA produced by stromal cells may regulate cancer cell invasion [29]. However, our results showed that there was no significant difference in uPA activity between different tissues. This may be attributed to the dynamic activity of uPA, which appears to be dependent on the particular cellular environments and states that it encounters [30].

## Conclusions

Our study shows that uPA system expression is significantly higher in peritoneal tissues of peritoneal metastasis patients than in nonperitoneal metastasis patients. This expression difference may provide a reference for diagnosis of peritoneal metastasis. However, there are still some limitations in this study. The number of cases included in this study was rather small, mainly because few patients with peritoneal metastasis were admitted in our hospital between July 2010 and December 2010. We tried to remove the influence of this problem by including tissues (including omentum majus, pelveoperitoneum, and diaphragmatic peritoneum) in this study. Further study with higher numbers of subjects is still needed to obtain clearer and more convincing results.

## Abbreviations

CEA: Carcinoembryonic antigen; DMEM: Dulbecco’s modified Eagle’s medium; ELISA: Enzyme-linked immunosorbent assay; GAPDH: Glyceraldehyde-3-phosphate dehydrogenase; PCR: Polymerase chain reaction; RT: Reverse transcriptase; RT-PCR: Reverse transcriptase polymerase chain reaction; uPA: Urokinase-type plasminogen activator; uPAR: Urokinase-type plasminogen activator receptor.

## Competing interests

The authors declare that they have no financial or nonfinancial competing interests.

## Authors’ contributions

YD and ZZ designed the study, and performed RT-PCR, ELISA, and activity detection with HZ and ZZhou. YD, MZ, and XW participated in the sequence alignment and drafted the manuscript. ZZ helped to conduct statistical analysis. All authors read and approved the final manuscript.
